# Density dependence and evolvability limit adaptive therapy in non-small cell lung cancer mouse model

**DOI:** 10.1038/s41598-026-63987-x

**Published:** 2026-08-04

**Authors:** Mariyah Pressley, Jessica J. Cunningham, Luiza Simoes, Robert A. Gatenby, Joel S. Brown, Stanislav Avdieiev

**Affiliations:** 1 Integrated Mathematical Oncology, H. Lee Moffitt Cancer Center and Research Institute, 12902 USF Magnolia Dr, Tampa, FL 33612 USA; 2 Department of Medical Engineering, College of Engineering, University of South Florida, Tampa, FL USA; 3 Fralin Biomedical Research Institute, Virginia Institute of Technology at Virginia Tech Carilion, Virginia Tech, 4 Riverside Circle, Roanoke, VA 24016 USA

**Keywords:** Non-small-cell lung cancer, Computational models, Experimental evolution, Cancer models, Targeted therapies, Cancer, Cancer therapeutic resistance

## Abstract

Using NSG mice grafted with H3122 non-small cell lung cancer cells, we compared outcomes from (1) continuous therapy with alectinib, (2) adaptive therapy where alectinib was cycled on and off based on a mouse’s tumor burden, and (3) no treatment. Adaptive therapy has proven successful in mouse models and clinical trials. The success of adaptive therapy improves when either there is density-dependent feedbacks favoring competition by sensitive over resistant cancer cells when therapy is off, or when the rate at which the population of cancer cells becomes more sensitive to therapy during periods of no treatment is faster than the rate at which resistance increases when on therapy. We found tumor growth rates were lowest under continuous therapy and highest under no therapy with adaptive therapy in between. We fitted the data to three separate game-theory models of resistance evolution where we let resistance be a quantitative trait consistent with resistance mechanisms to alectinib. All models successfully identified why adaptive therapy proved less successful than continuous: there was no evidence for favorable density-dependent feedbacks; and there was no significant difference in the rate of evolution towards or away from resistance when therapy was on or off.

**Supplementary Information:**

The online version contains supplementary material available at https://doi.org/10.1038/s41598-026-63987-x.

## Introduction

Lung cancer remains the leading cause of cancer-related death with non-small cell lung cancer (NSCLC) comprising 81% of diagnoses^1^. Approximately 5% of NSCLC patients harbor oncogenic ALK fusion genes^2^. Molecular targeted therapy with tyrosine kinase inhibitors (TKIs) is first-line therapy for patients with ALK rearrangements^3^. Although targeted therapy has improved survival rates^4,5^, resistance mechanisms invariably lead to disease progression^6^. Drug resistance and recurrent disease have led to the development of multiple generations of ALK inhibitors^6–8^. Unfortunately, once the disease progresses, patients become resistant to the next drug, and so forth. Thus, overcoming resistance remains a challenge for long-term disease control with TKIs.

Adaptive therapy (AT) is a therapeutic strategy that aims to delay or prevent cancer progression due to resistance. Traditional cancer treatment strategies often employ maximum tolerated dose (MTD) in which a high, static dosage is administered over a fixed period. In comparison, adaptive therapy outlines a set of rules that instruct what to do based on changes in tumor burden in response to treatment. This allows the physician to adapt the frequency of therapy, dosage given, or type of therapy based on the patient’s tumor burden^9^.

Every step of tumorigenesis from initiation through progression is an evolutionary event^10–13^. Cancer cells are continuously under selection to enhance their survivorship and proliferation given their circumstances within diverse tumor microenvironments. This leads to a tumor population of diverse cell phenotypes. A cell’s ability to survive and proliferate is directly influenced by its phenotypic traits and environmental conditions. Anticancer drugs introduce a new selective pressure into their environment that ideally cancer cells fail to overcome. However, tumor heterogeneity and the cancer cells adaptations pose a challenge to traditional treatment strategies^14^. When resistant cells exist prior to therapy then anticancer drugs are only successful at eliminating sensitive cells. On the other hand, exposure to anticancer drugs can allow cells to evolve resistance^9,15^. Either way, the standard treatment paradigm selects for an entirely resistant tumor cell population if it cannot swiftly eradicate all cancer cells. Therefore, in many instances, like NSCLC, the administration of therapies at MTD may not be the most effective way to combat cancer as an evolutionary process.

Adaptive therapy offers an alternative strategy designed to maintain a stable, treatable (responsive) tumor burden. The feasibility and benefit of adaptive therapy have been supported by various mathematical models^9,16–18^. The effectiveness of this strategy has been validated in several contexts. In an in vivo study of adaptive therapy, authors showed the feasibility of the method by successfully controlling tumor burden in an ovarian cancer mouse model^9^. Pedro et al. demonstrated tumor control in a pre-clinical breast cancer model by adapting the dosage to changes in tumor burden^19^. In a melanoma mouse model, an adaptive protocol outperformed continuous and intermittent therapy^20^. Most notably, a clinical trial of adaptive therapy for patients with metastatic castrate-resistant prostate cancer (mCRPC) significantly extended progression-free survival compared to MTD^21^. The success of adaptive therapy in various cancer models inspires our exploration of its potential to combat resistance in NSCLC.

In the present study, we utilized an AT protocol in an H3122 NSCLC mouse model. Unexpectedly, the results of our in vivo study showed inferiority of AT compared to continuous therapy. This prompted a further investigation into why and when AT would be inferior and what, if anything, could improve its efficacy. We analyze the results of an AT trial in an NSCLC preclinical model by mathematically modeling the evolution of resistance. We allow the experimental data to guide the development of three models, each with a different method for how treatment may suppress cell proliferation. Each model is a fitness generating function, *G*-function, which allows us to examine the evolutionary dynamics behind the treatment results. We calibrated the models to longitudinal volumetric data from the two therapeutic arms of the preclinical trial: continuous therapy and AT. Multiple iterations of calibrations were performed to determine mouse-wide and mouse-specific parameter values. After determining the best fit parameter values, we simulated “what-if “ scenarios to investigate other possibilities for optimizing treatment.

## Results

### Continuous therapy extends survival in NSCLC model

**Fig. 1:**
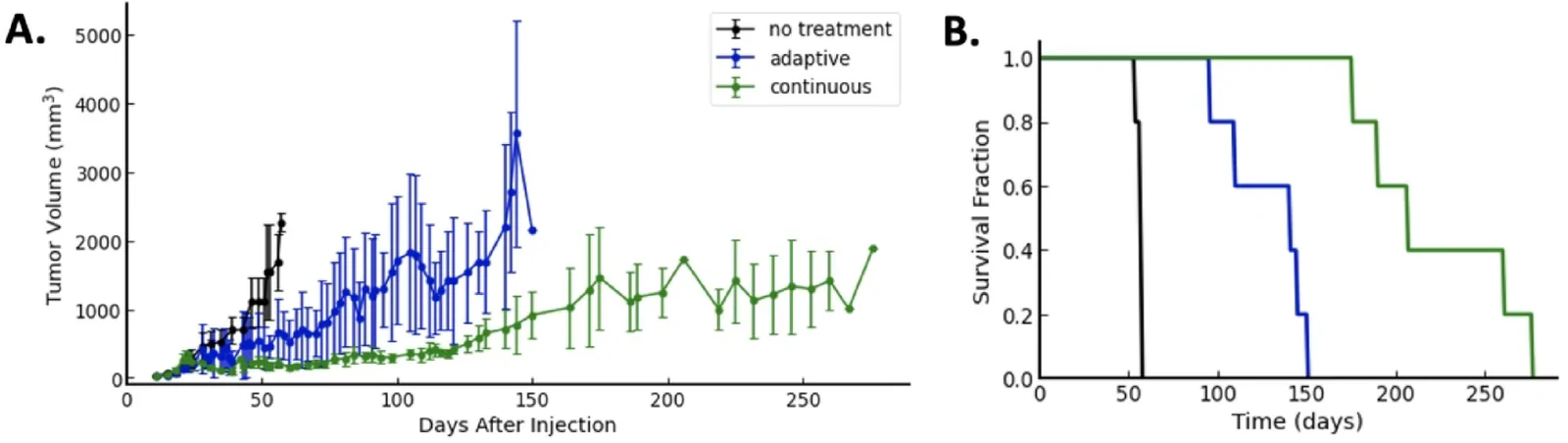
AT in an in vivo NSCLC model is less beneficial than continuous therapy. (A) Tumor volume changes over time in three preclinical trial groups: control (no therapy), AT, and continuous therapy. Data is shown as the mean ± standard deviation. Continuous therapy maintains a lower tumor burden for a longer duration than AT. (B) Survival fraction of mice in each therapy arm. The survival fractions are significantly different (Kruskal-Wallis, *p <* 0.002).

In this study, we evaluated the effectiveness of TKI, alectinib, in a preclinical NSCLC mouse model, under different therapeutic protocols: continuous therapy and AT. In comparison to the control group where no treatment was given, alectinib elicited a favorable response in both treatment groups. However, this response was limited as neither treatment strategy maintained a survivable stable tumor burden nor prevented eventual tumor growth. Mice that received no treatment exhibited the shortest average overall survival of 52 days. Adaptive application of the drug led to a rapid increase in tumor burden resulting in an average overall survival of 127 days. Mice treated continuously survived significantly longer, with an average overall survival of 221 days (Kruskal-Wallis test, *p <* 0.002) (Fig. 1). This contradicted our initial hypothesis that AT would yield the most favorable response, prompting us to further investigate the underlying factors influencing therapeutic outcomes. We turned to mathematical modeling to uncover potential mechanisms that led to the observed outcomes and identify opportunities to improve treatment efforts.

### Lack of evidence of density-dependent feedback

**Fig. 2:**
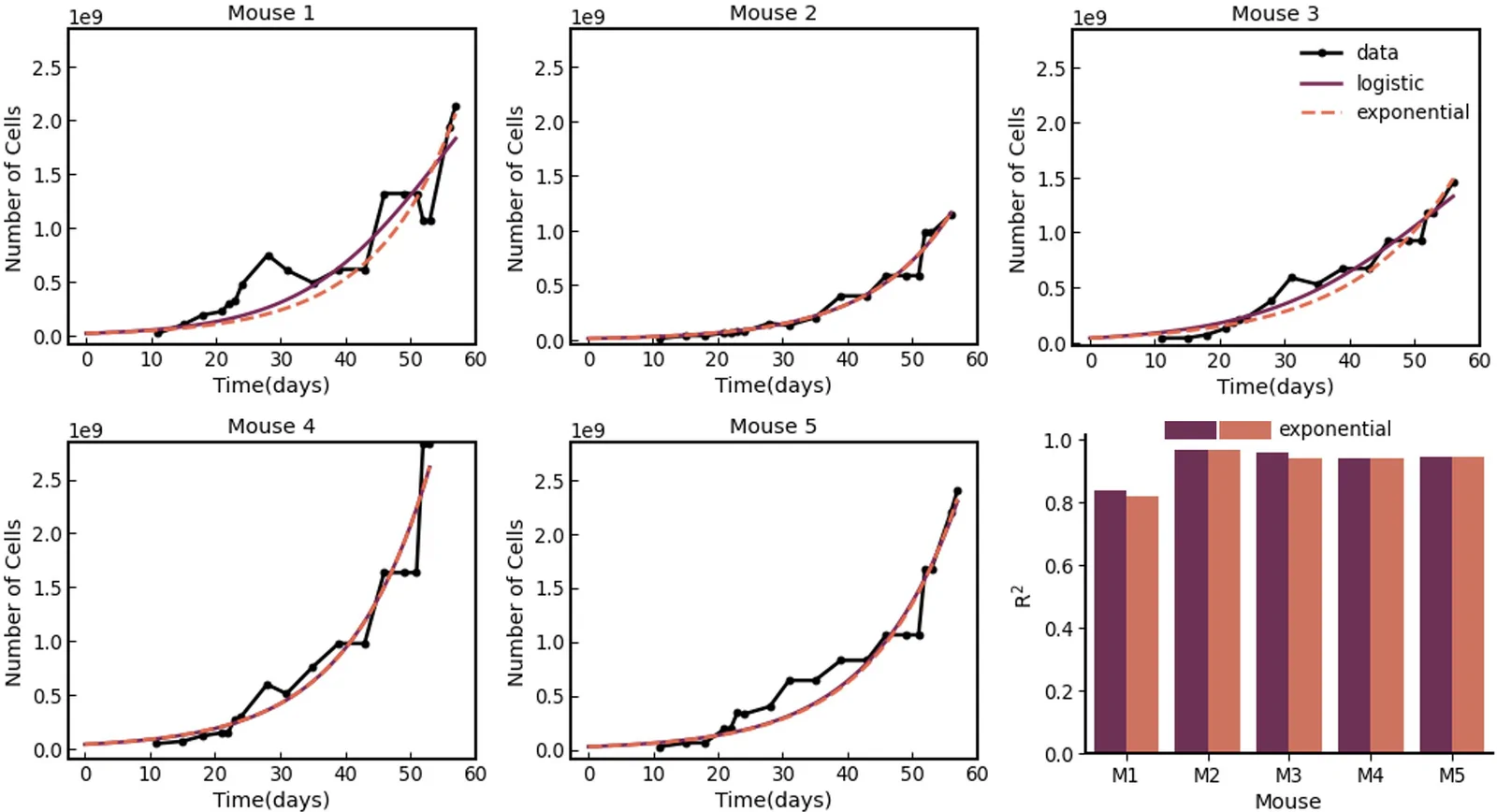
Exponential and logistic growth models adequately capture tumor growth over time in the absence of therapy. Both models were fit to the longitudinal tumor volume data converted to number of cells. *R*^2^ scores were one metric used to determine the quality of fit of tumor growth data from mice (*n* = 5) that were not treated. For each mouse, *R*^2^ ≥ 0.8 for all model fits, indicating that all models effectively capture the tumor growth dynamics.

Starting with the control mice we compared exponential growth (no density dependence, just an intrinsic growth rate, *r*, in units of per day) with logistic growth (density dependence introduced by having a carrying *K*, units of cell number). Several metrics were used to evaluate evidence for density-dependence, and whether exponential or logistic growth best recapitulates the tumor growth dynamics of the control mice (Fig. 2). First, we compared the coefficient of determination (*R*^2^). For each mouse, both models yielded high *R*^2^ values. The logistic model by virtue of having an extra parameter always returned an equal or slightly greater *R*^2^ value. The addition of an extra parameter (*K*) did little to improve the model’s overall fit. To further explore if the inclusion of *K* is supported, we use the Akaike Information Criteria score (AIC). The AIC score quantifies the quality of model fit while taking into consideration the number of parameters used in the model. This balances the tradeoff between goodness-of-fit and number of parameters to select the best model. Comparing AIC scores shows that the exponential model is more parsimonious descriptor of the data than the logistic model for all mice except one (Table 1).

**Table 1:** Estimated growth parameters for exponential and logistic growth models.

Mouse	Exponential *r*	Logistic *r* & K	∆AIC
1	0.080	0.092 3.3*10^9^	− 0.75
2	0.079	0.080 9.6*10^10^	− 2.91
3	0.064	0.076 2.4*10^9^	4.14
4	0.080	0.081 1*10^11^	− 3.10
5	0.078	0.079 2.84*10^10^	− 2.76

To compare the goodness of fit between the two models, we calculate ∆*AIC*, which represents the difference between the AIC of the exponential model and the AIC of the logistic model. If ∆*AIC* is negative, it suggests that the exponential model provides a more parsimonious fit to the data. Moreover, a ∆*AIC* value smaller than − 2 indicates that the difference is significant.

We opt for the exponential growth model over logistic growth in what follows for several reasons. First, for the exponential model and logistic model *R*^2^ ranged from 0.816 to 0.964 and 0.835–0.964, respectively. Second, only control mouse 4 suggested a better fit with logistic. Third, the estimates of *K* are wildly different across the mice whereas those for *r* are not (Table 1). Finally, in all cases a model fit with density-dependent population growth still shows an accelerating curve of population size versus time for the actual data, suggesting growth is still roughly in its exponential phase. Furthermore, for subsequent analyses with continuous and adaptive therapy drug treatments, we reduce the number of mouse-specific parameters by fixing the value of *r* = 0.076 reflecting the mean value of the 5 control mice.

### Models recapitulate tumor growth dynamics

Based on exponential tumor growth in the absence of therapy, we crafted three *G*-functions that include the effect of therapy, *m*, and a resistance trait, *u*. A G-function permits the simultaneous modeling of ecological (changes in tumor burden, *x*) and evolutionary (changes in the cancer cells’ resistance trait, *u*) dynamics^22,23^. For modeling eco-evolutionary dynamics, this modeling formulation is like that of adaptive dynamics^24,25^. The function *G*(*v*,*u*,*x*) describes the expected per capita growth rate of a focal individual with trait *v* in a population comprised of individuals with trait *u* with a population size of *x*. For our purposes *v*, *u* and *x* are non-negative continuous variables.

With this formulation the change in population size is given by $\:\frac{dx}{dt}=xG$ evaluated at *v* = *u* namely where the trait of the focal individual is set equal to that of the population. The evolutionary dynamics conform to Fisher’s Fundamental Theorem of Natural Selection. The adaptive landscape plots *G* versus *v*, the expected fitness to a focal individual. The strategy of the population, *u*, evolves towards increased fitness, scaled by an evolutionary speed term (σ) such that $\:\frac{du}{dt}=\sigma\:\frac{dG}{dv}$ where $\:\frac{dG}{dv}$, the slope of the adaptive landscape, is evaluated at *v* = *u*. In this manner evolution by natural selection responds to fitness benefits from a unilateral change in strategy by an individual of the population, not by how fitness would change with a collective change in strategy by the entire population, $\:\frac{dG}{du}$. This distinction becomes important when an individual’s fitness is directly influenced by the strategy present in the population, *u*. In the subsequent models, this will not be the case. *G* will simply be a function of *v* and possibly *x*; in which case there is no difference between setting *v* = *u* and evaluating $\:\frac{dG}{du}$ versus evaluating $\:\frac{dG}{dv}$ and then setting *v* = *u*. For notational completeness we will maintain the distinction between a focal individual’s strategy, *v*, and that of the entire population, *u*^22^.

The speed term σ in classic quantitative genetics represents trait heritability or additive genetic variance. For cancer cells this applies but higher values may reflect epigenetic changes and still higher values phenotypic plasticity. If non-zero in our experiments, σ scales the capacity of the cancer cells to adjust their level of drug resistance in response to selection for resistance. The combined population and strategy dynamics represent the eco-evolutionary dynamics of the system.

We choose to model resistance to therapy as a continuous trait. Other models of therapy resistance consider a sensitive and resistant population^26^ or even a hybrid form^16,27^. While it remains an empirical question, continuous traits such as upregulating efflux transporters (over-expression of P-glycoproteins) or upregulating binding proteins are common resistance mechanisms for cancer cells faced with tyrosine kinase inhibitors including alectinib^28^.

The following three *G*-functions vary in terms of the functional form for how a focal cell’s resistance trait, *v*, reduces drug efficacy. What they have in common is exponential growth in the absence of therapy and a cost of resistance by which the intrinsic growth rate, *r*, declines linearly as resistance increases. Each of these forms ensures that *v*, and by extension the population’s trait value *u* takes on values between 0 and < 1:

4 $$\:\mathrm{M}\mathrm{o}\mathrm{d}\mathrm{e}\mathrm{l}\:1:G\left(v\right)=r\left(1-v\right)-\:\frac{m}{1+bv}$$

5 $$\:\mathrm{M}\mathrm{o}\mathrm{d}\mathrm{e}\mathrm{l}\:2:G\left(v\right)=r\left(1-v\right)-\:{me}^{-bv}$$

6 $$\:\mathrm{M}\mathrm{o}\mathrm{d}\mathrm{e}\mathrm{l}\:3:G\left(v\right)=r\left(1-v\right)-\:{m(1-v)}^{b}$$

In each model, a focal cell’s fitness is directly influenced by its strategy, *v*, but not by the population’s trait value, *u*, or population size, *x*, as can occur in more complicated *G*-functions with frequency- and density-dependence^29^.

**Fig. 3:**
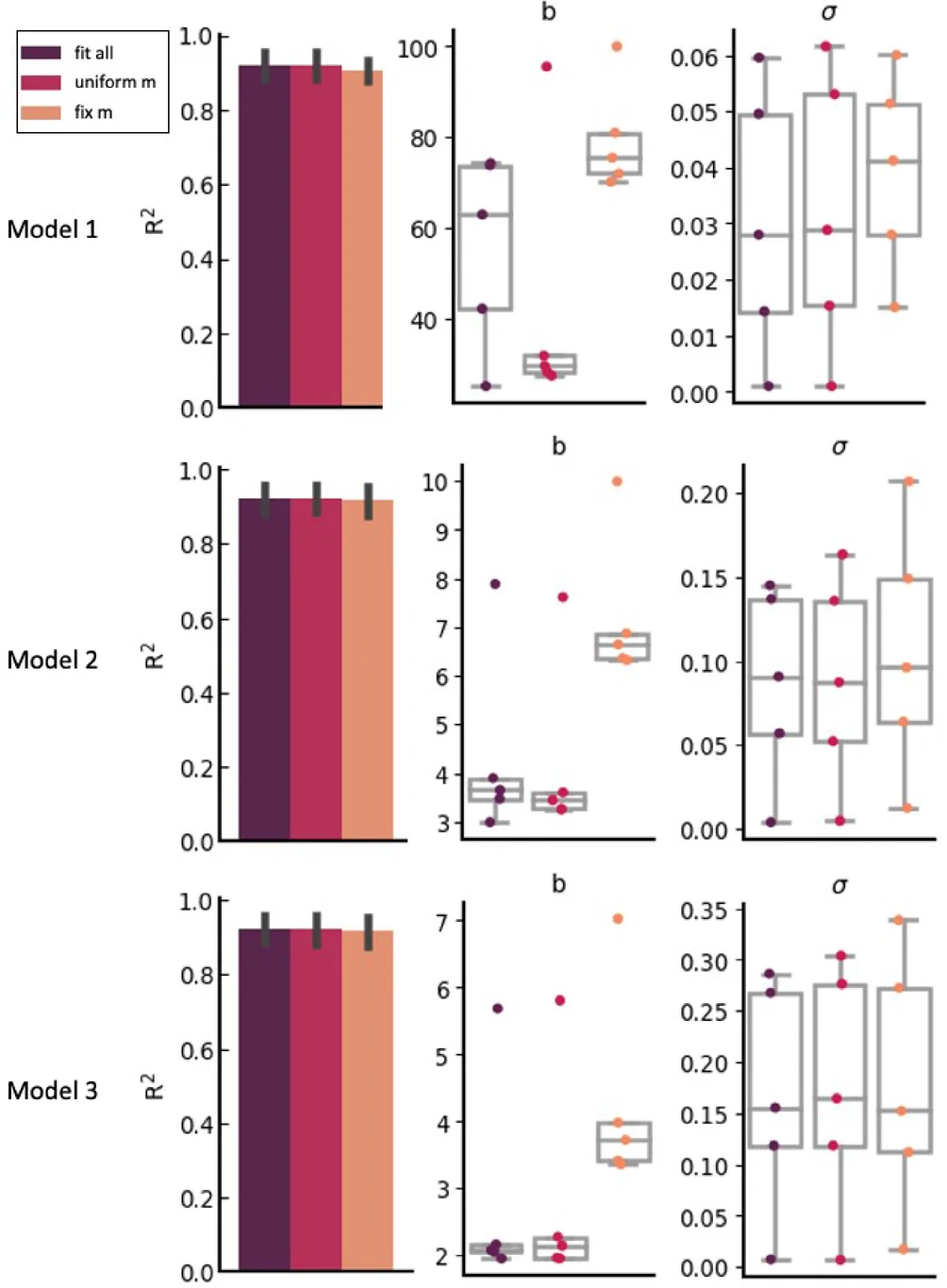
Fitting each model to the continuous therapy group data demonstrates that data can be described assuming maximum drug efficacy (*m* = 1). Models 1, 2, and 3 are fit to longitudinal data converted to number of cells for each mouse in the continuous therapy group using multiple approaches (fit all, uniform *m*, and fix *m*). The left panel shows a comparison of the descriptive ability of each model. The average *R*^2^ scores and standard error are shown. The middle panel shows a comparison of the distribution of estimated values of the benefit of resistance (*b*) based on model fits. The right panel shows a comparison of the distribution of estimated values of the evolutionary speed (*σ*) based on model fits.

Each of the three models represent a monomorphic population where a single evolutionary strategy, *u*, defines the entire tumor cell population. In this case, the focal cell strategy is the only strategy available within the population such that *v* = *u*. Utilizing how the tumor size changes over time, each model was fit to data from mice in the continuous treatment group (*n* = 5) to identify the model that most accurately represents the data (Fig. 3). For simplicity, we assume therapy was applied continuously, despite the weekend holidays. Given that the drug was applied daily except for Saturdays and Sundays with tumor measurements on Tuesdays and Fridays, one can imagine using a pharmacokinetics (PK) model that sees effective drug dosage, *m*, spiking with each dose and then decaying prior to the next dose. In fitting the data for continuous therapy, the PK model produces greater day-to-day variation in tumor growth and a weekend effect, it did not provide an overall better fit than the smoother fit achieved by assuming a constant value for *m*.

Next we moved to fit the continuous therapy mice. We fixed the growth rate, *r*, to the modal estimate from the control mice (*r* = 0.076 per day, leaving three remaining parameters for fitting the tumor dynamics (*m*, *b*, and *σ*). We then examined fits using three approaches that placed progressively greater restrictions on drug efficacy, *m*:

1. Fit All: In our first approach, we allow the algorithm to find the best fit values for all parameters (*m*, *b*, and *σ*) specific to each mouse.
2. Uniform *m*: This approach is the same as the first except the value of *m* is optimized across all mice. This approach returns a single value of *m* for all five mice.
3. Fix *m*: Lastly, we fix *m* equal to 1 and the fitting algorithm returns mouse-specific values for *b* and *σ*. With the global value of *r* = 0.08 per day this gives the drug a high initial efficacy as for all three models *G* = 0.08–1 = -0.92 per day at *v* = 0 resulting in a 60% drop in the cancer cell population after 24 h: e^− 0.92^ = 0.4.

Our results show that each model can recapitulate the tumor growth dynamics during treatment, regardless of the specific approach used for fitting. In each approach, the quality of the fit is virtually indistinguishable for most mice (Fig. S1). Differences between model fits are shown in the parameter distributions. With fitting approach 1, where all parameters are fit freely, the mouse-specific values for *b* (benefit of resistance) obtained from model 1 exhibit a greater variability than the other two models. Also, model 1 selects values for *m* and *b* that are greater than those selected by models 2 and 3.

Under fitting approach 2, the mouse-wide value of *m* given by model 1 (*m* = 0.41) is at the lower end of the range of values selected when *m* was mouse-specific in fitting approach 1. Models 2 and 3 selected mouse-wide values for *m* (*m* = 0.14, *m* = 0.13 respectively) equal to the average mouse-specific values of *m* from approach 1. In terms of *σ* (rate constant for phenotypic change in response to selection), model 1 exhibits the smallest range of values, while model 3 shows the largest.

Under fitting approach 3 (*m* = 1 for all mice and models), there are corresponding increases in the estimated value of *b* compared to the other approaches. However, the estimated values of *σ* remain similar between fitting approach 2 and 3. Because of the higher *m*, approach 3 initially returns lower estimates of cell numbers than approach 2. Thereafter, with the evolution of higher values of resistance, where higher values of *u* are more effective with approach 3 than 2, there is rapid convergence in population size estimates. This is to be expected as estimated values for *m* under approaches 1 and 2 were always less than 1, and substantially so for models 2 & 3. For each model, a lower value of *m* (mouse-wide *m*) coincides with lower values of *b*, and a greater value of *m* (*m* = 1) coincides with greater values of *b*.

We can use fixed values for *m*, *b* and *σ* derived from the continuous therapy mice to see how well the model fits the adaptive therapy cohort of mice. To do so we used values from approach 3: *m* = 1, with *b* and *σ* set to the mean for the five mice. By choosing *m* = 1, instead of estimates from 1 to 2, we ensure the drug is initially very effective (similar results occur even if *m* is set to lower values).

To fit the 5 mice treated with adaptive therapy, we used approach 3, setting m = 1, *r* = 0.076 for all mice and letting b and σ be mouse specific. By choosing m = 1, instead of estimates from 1 to 2, we ensure the drug is initially very effective. All three drug-efficacy models overall provide good fits for the data (Fig. S2). Most striking is how each model formulation gives near identical fits save for mouse 14 where models 1 & 3 match the early data point but overestimate tumor size over most of the time series. For mouse 14, model 2 provides a better overall fit, while providing a poorer estimate of the first 4 tumor volume measures. Collectively, none of the model fits were able to successfully track the magnitude of the on and off drug cycles. The best models fit simply smoothed over the ups and downs of tumor volumes for each of the mice. Trying lower values of m such as values from Approach 2 had little effect on the fits and did not alter the results from fitting the AT mice.

### In silico trials corroborate experimental results

**Fig. 4:**
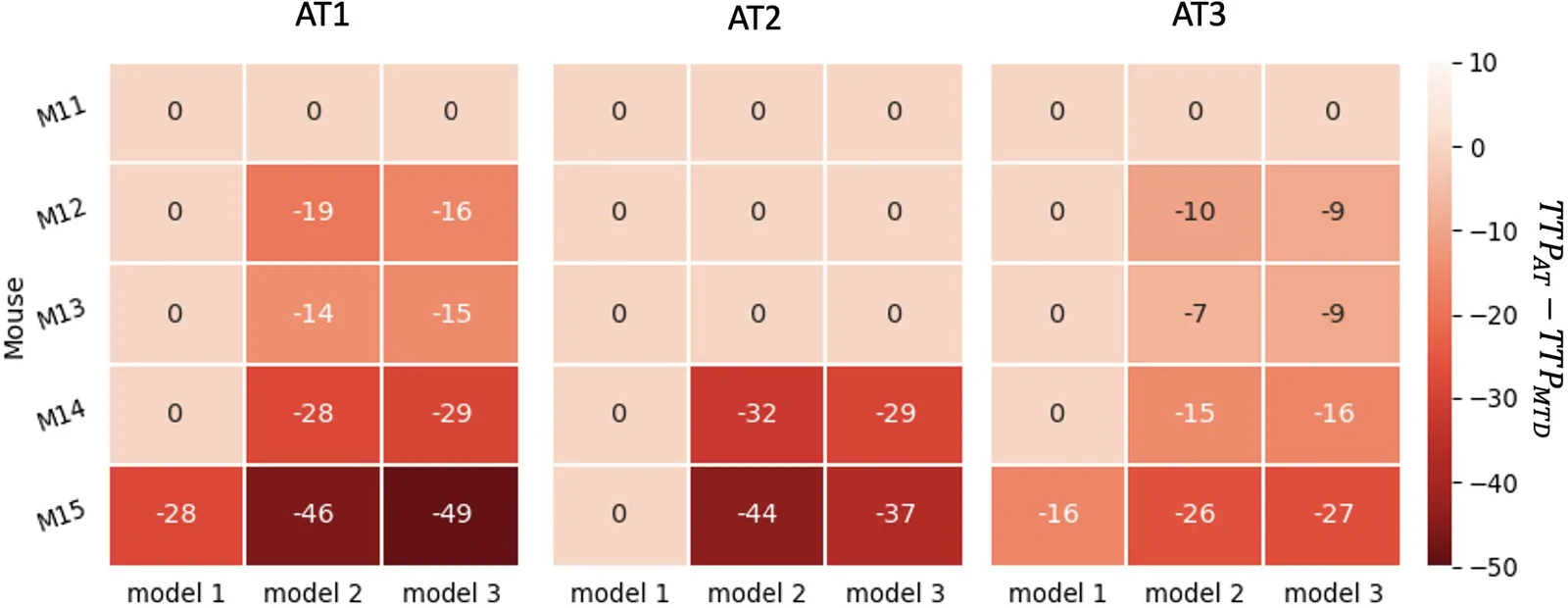
The difference in time to progression (TTP) between each simulated adaptive therapy (AT) protocol and continuous maximum tolerable dose (MTD) varied between models, mice, and AT protocols. Heatmaps represent the difference in TTP between a particular AT protocol and continuous therapy. Tumor growth progressed earlier on AT protocols compared to continuous therapy. Of the AT protocols, AT3 (the experimental protocol) extended TTP the longest. The zeros indicate combinations of mouse, model and AT protocol where adaptive therapy never drove the tumor burden low enough to cycle off therapy, thus resulting in continuous MTD therapy by default and resulting in no difference.

We investigated whether adaptive therapy might have been effective for cohort of mice receiving continuous therapy. To do this, we considered each mouse and each drug-efficacy model using the mouse’s parameter estimates for *b* and *σ* from approach 3. We asked whether alternative treatment strategies could have been more effective. We conducted an in silico trial where three AT protocols were simulated for each mouse to discover what might have happened had a mouse received a different adaptive-therapy treatment regimen (Fig. 4). Each AT protocol is as follows:

1. AT1: Drug is administered at maximum efficacy (*m* = 1) until the tumor cell population size decreases by 25%. Treatment is then discontinued (*m* = 0) until the tumor recovers to its initial population size. This is similar to the experimental protocol, except the tumor is allowed to regrow to its initial size.
2. AT2: Drug is administered at maximum efficacy (*m* = 1) until the tumor cell population size decreases by 50%. Treatment is then discontinued (*m* = 0) until the tumor recovers to its initial population size. This protocol was adapted from Zhang et al.^30^.
3. AT3: Follows the experimental protocol.

Model 1 simulations indicate that AT would only have been feasible for a single mouse as all other mice failed to demonstrate a reduction in tumor size ≥ 25%. In the mice where AT was performed, only two adaptive protocols were simulated because tumor size did not decrease by at least 50%. Both models 2 and 3 were able to simulate AT1 and AT3 protocols for the majority of mice, however, the AT2 protocol could only be simulated for two mice due to the aforementioned reason (Fig. S3).

We assessed treatment performance by evaluating time to progression (TTP). This is defined as the time when tumor size exceeds its initial size at the start of treatment. Consistent with experimental findings, the in silico trial corroborated the inferiority of the adaptive experimental protocol (AT3) to continuous treatment. Furthermore, no adaptive protocol was able to extend TTP longer than continuous therapy. For each virtual subject, we determined the time loss by AT relative to continuous therapy by subtracting the TTP under continuous therapy from the TTP under AT.

According to our mathematical models, AT1 was predicted as the least effective treatment strategy, leading to progression the fastest. When AT2 could be simulated (models 2 and 3 for mice 14 and 15) it, like AT1, performed more poorly than AT3. The effectiveness of AT exhibited more variation among mice than among models. However, this observation should be interpreted with caution due to the limitations of model 1 in simulating AT. Our results show that models 2 and 3 result in roughly the same estimates for TTP for each mouse under a specific treatment protocol. Model 1 could only simulate AT1 and AT3 for mouse 15, predicting the greatest TTP for each protocol compared to the other models. Across the board, parameterization for mouse 15 elicits the greatest difference in TTP between treatment strategies.

### The adaptive landscape

**Fig. 5:**
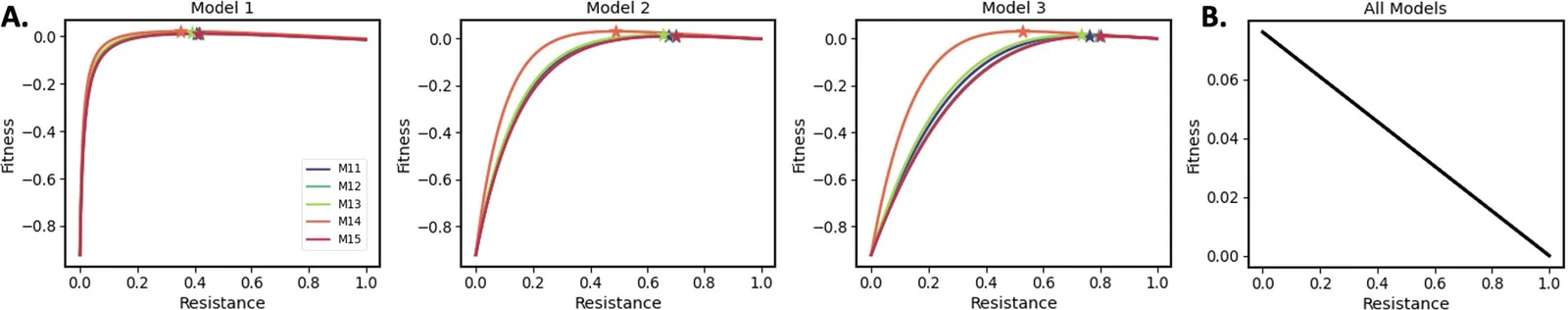
The adaptive landscapes for the five mice under continuous therapy setting *m* = 1, *r* = 0.076 and the values for *b* and σ to the mouse-specific estimates. The adaptive landscape plots per capita growth rate, *G*, as a function of a focal cell’s resistance strategy, *v*. Stars denote peaks of the respective landscapes. The adaptive landscape depicts a greater cost of not being resistant when therapy is on than being resistant when therapy is off. The slope of an adaptive landscape determines the rate at which fitness changes. (**A**) The three panels show the adaptive landscapes for the three models of drug efficacy when therapy is on. Note the asymmetric form where having too little resistance as a cancer cell is worse than having too much. The steeper slope at low levels of resistance indicates a more rapid change in fitness. (**B**) When therapy is off, the adaptive landscape for each model is the same. The constant slope indicates that the rate of change of fitness does not depend on the current resistance level.

To further our investigation of why drug holidays with adaptive therapies did not improve TTP, we can examine the adaptive landscape (Fig. 5) in the presence or absence of therapy. The adaptive landscape shows fitness (*G*) as a function of the resistance strategy (*u*). Because of the exponential growth model, *G* is not a function of population size, the shape of the landscape with or without therapy remains the same despite changes in tumor size over time. However, the adaptive landscape looks different in the presence and absence of therapy. In the absence of therapy, the optimal level of resistance is u* = 0. In the presence of therapy, the fitness peak occurs at 0 ≤ u* < 1 because the cost of resistance scales as $\:r\left(1-v\right)$ where *v* = 1 represents maximal resistance beyond which the cancer cells would simply die off regardless of therapy. Interestingly, it is possible to solve for u* as a function of *r*, *m* and *b* by solving for $\frac{{\partial G}}{{\partial \upsilon }}$ = 0. Thus, for each of mice, for the three models and for the three approaches it is possible to solve for u* and draw the case-specific adaptive landscape. For all cases when therapy is on, the adaptive landscape is asymmetric around the fitness peak – steeper to the left than to the right. This implies a large cost in fitness to not be resistant in the presence of therapy. In other words, the greater the slope, the higher the penalty for not adopting the most advantageous strategy in terms of fitness. This also indicates that small changes at lower levels of resistance will result in large changes in fitness. When comparing models, we see that model 1 produces an adaptive landscape with the steepest slope when u < u*. Therefore, this model predicts evolution to happen at a faster rate than models 2 or 3. In all cases, with time, under continuous therapy each mouse should evolve to its u*.

When therapy is off, the adaptive landscape is linear with a negative slope equal to *r* and maximum fitness at u* = 0. This is the same for each model and mouse because the growth dynamics are the same and *r* is a fixed value. This showcases the minimal cost of being resistant in the absence of therapy. In contrast to the adaptive landscape with therapy, we see that changes in an individual’s strategy, regardless of resistance level, result in a uniform change in fitness. Put together, when therapy is applied and u < u*, *u* increases. When therapy is withdrawn the current strategy is now placed on a different adaptive landscape and *u* decreases. The rate at which *u* changes is determined by ($\frac{{\partial G}}{{\partial \upsilon }}$) scaled by the evolutionary speed term, *σ*. Because our models assume evolutionary speed to be the same with or without therapy, the rate of forward and backwards evolution depends only on the slope of the adaptive landscape. Our results show that evolution is highly favored towards increasing resistance when therapy is administered but not inclined to decrease as fast when therapy is withdrawn. Thus, forward evolution in the presence of therapy is faster than the reverse in the absence of therapy. This will work against the efficacy of adaptive therapy.

### Variable rates of evolvability improve adaptive therapy

We modified the models to have two distinct evolutionary speed terms, one for when therapy is administered and withdrawn, *σ*_*on*_ and *σ*_*off*_, respectively. Simulations of adaptive protocols were rerun where *σ*_*on*_ remained the same as previous values determined by model calibration and *σ*_*off*_ was 2, 3, 5, and 10 times that of *σ*_*on*_. This was only performed with models 2 and 3 because model 1 was insufficient for examining AT. Also, we only look at AT1 and AT2 protocols for similar reasons. Altering the speed of evolution in these cases would be futile as the tumor burden never reaches an appropriate size for therapy to be withdrawn.

**Fig. 6:**
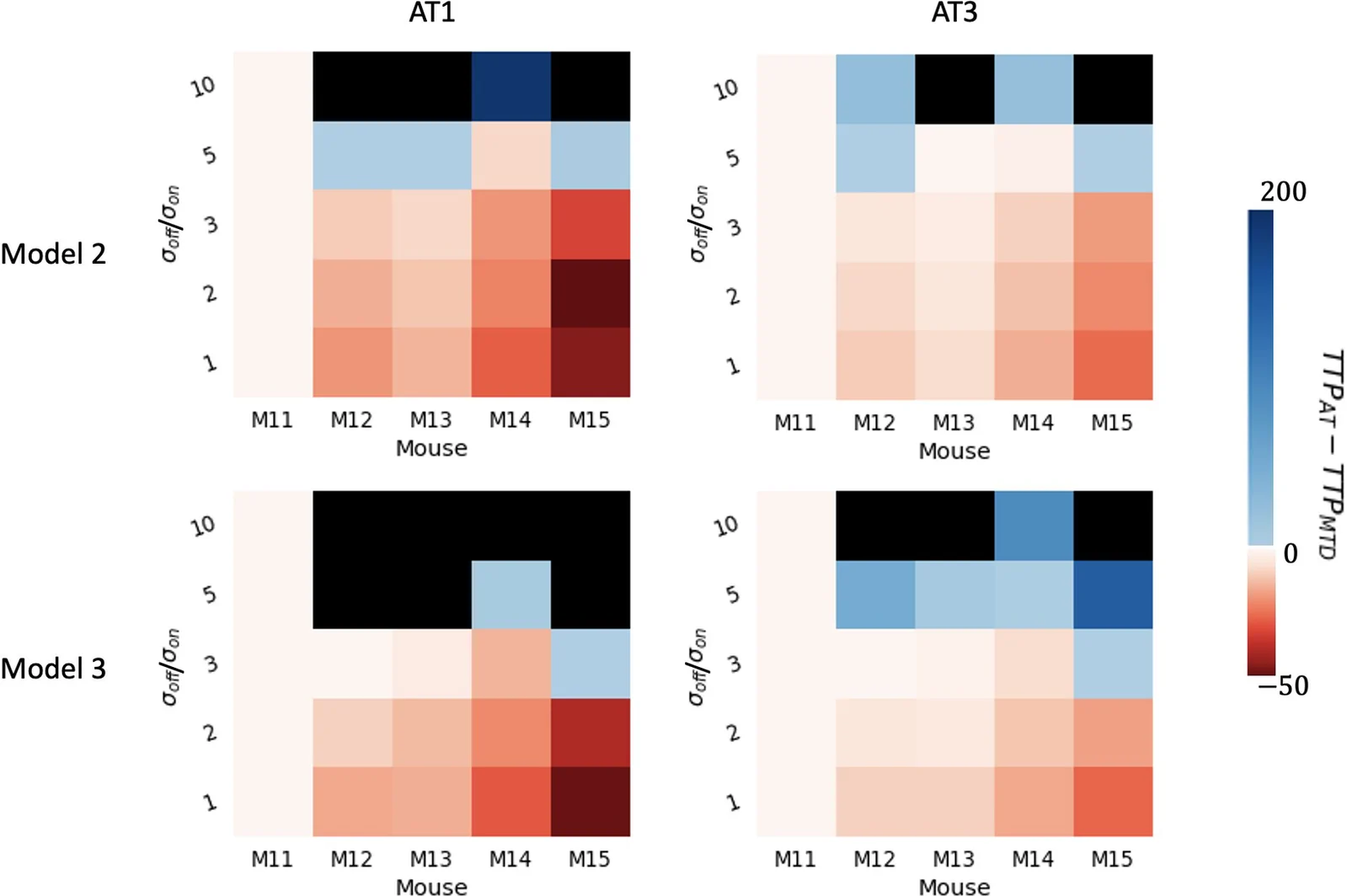
Increasing the speed of evolution in the absence of therapy presents an opportunity to improve AT. Heatmaps of the difference in TTP between adaptive protocols and continuous therapy at varying ratios of $\frac{{{\sigma _{off}}}}{{{\sigma _{on}}}}$ where the speed of evolution when therapy is on (*σ*_*on*_) is held constant and the speed of evolution when therapy is off *σ*_*off*_ is increased. Red and blue shaded boxes are when tumor growth during AT progresses before and after continuous therapy, respectively. Black boxes are when the tumor size did not progress within the simulated time (500 days).

Results demonstrated that increasing the speed of evolution in the absence of therapy can increase the efficacy of AT. Figure 6 shows a general trend where faster speeds of evolution when therapy is off increases the difference in TTP between adaptive and continuous therapy. We see that AT is most beneficial at larger fold changes ($\frac{{{\sigma _{off}}}}{{{\sigma _{on}}}} \geqslant$ 5). In several scenarios, there was no progression during the time simulated (500 days). Model 3 predicts a greater benefit of AT at larger fold changes than model 1. When the TTP of adaptive protocols surpasses that of continuous, AT1 delays progression the longest. This is the opposite of simulations where *σ*_*on*_ = *σ*_*off*_, resulting in AT1 leading to the quickest TTP.

## Discussion

In this study, we investigated the ability of AT using alectinib to prolong efficacy of targeted therapy in NSCLC. Alectinib is a highly selective, next-generation ALK tyrosine-kinase inhibitor that binds the ALK fusion oncoprotein and shuts down downstream pro-survival signaling (e.g., STAT3/AKT), leading to growth arrest and apoptosis in ALK-rearranged NSCLC cells. It retains activity against several crizotinib-resistance mutations (including the gatekeeper L1196M)^31,32^. Multiple human trials have confirmed first-line efficacy of alectinib^5,33–36^. To our knowledge, alectinib has not yet been tested in a clinical adaptive/evolutionary trial, however few reports exist on alectinib in this space using preclinical models, including evolutionary-game assays and recent antifragility modeling. Notably, Kaznatcheev et al. (2019) in a set of co-culture experiments showed that fibroblasts and alectinib can switch the evolutionary game between sensitive and resistant NSCLC cells - key conceptual support for evolutionary scheduling^37^. West et al. (2025) propose that the shape (convex/concave) of the alectinib dose-response in mice predicts whether fluctuations (i.e., intermittent/adaptive schedules) would help or harm^38^. Although AT has shown promise in extending progression-free survival in some clinical settings, our results demonstrate, contrary to our hypothesis, AT was not a beneficial alternative treatment strategy in targeted therapy for NSCLC. Instead, we found continuous therapy was more effective in suppressing tumor growth. This divergence from earlier successes highlights that the effectiveness of AT is highly context-dependent and need to incorporate specific tumor ecology and evolvability of each individual tumor ecosystem.

This extended the aim of our study to investigate why AT failed to perform better than continuous therapy. Through mathematical modeling, we can identify 3 reasons for the observed results. First, the absence of density dependent feedbacks that can accelerate drug resensitization when therapy is off whereby competition among cancer cells favor more sensitive cells due to a cost of resistance. Secondly, we find the rate of evolution towards sensitivity was slower than the rate of evolution towards resistance. And third, the adaptive landscape when therapy is on is asymmetric around the optimal level of resistance. Around the optimum having too little resistance is worse for the cancer cell than having too much.

The underlying success of AT requires prevention of competitive release, a phenomenon in which removal of a dominant competitor species “releases” resources that allows a less-dominant species to survive and grow^30,39–41^. In the context of cancer, the less dominant species are resistant cells that, in the absence of treatment, are less fit due to the cost of resistance. However, when an anti-cancer drug eliminates the dominant population of treatment-sensitive cells, it also removes the competition the previously suppressed proliferation of the resistant cells. AT introduces a variation in selection pressure through drug holidays or modulation, allowing one to suppress resistance by maintaining a population of sensitive cells.

In modeling, competition is framed as density-dependent growth such that as the population size increases the fraction of cells dividing decreases. Several models have been developed to compartmentalize resistant and sensitive cells and investigate their competitive interactions in a density-dependent manner^16,17,30,37,42,43^. Perhaps the most common modeling approach uses Lotka-Volterra competition models^16,17,30,43^. The mCRPC clinical trial was preceded by such a model, where the total tumor burden was comprised of distinct cell populations, and the magnitude of competition between cell types was explored through competition coefficients^30^. A retrospective analysis of the clinical trial identified that a greater competitive effect of sensitive cells on resistant cells than vice versa led to the efficacy of AT^21^. Other forms of density-dependent modeling have also identified the potential benefit of AT^42,44^.

In comparison, our model demonstrates growth in the experimental tumor is not influenced by population density and has no competitive interactions. The growth of a population is governed by two principles: (1) under constant conditions, populations grow exponentially and (2) conditions are never constant, so there are limits to growth^45^. In the context of cancer, the tumor microenvironment is constantly changing due to the presence or absence of nutrients, normal cells, and anticancer drugs^46^. Although modeling tumor growth as exponential may seem futile given these complexities, studies show that in vivo tumor growth models can exhibit exponential growth dynamics^47^. In fact, models depicting saturating growth dynamics, begin with an exponential growth phase, during which competition between subpopulations is minimal. In this study, the lack of evidence supporting density dependence does not imply unlimited population growth but rather suggests that negative feedback from density dependence is not yet realized. Future density-dependent feedback may have occurred, but it remained negligible in the time frame of our experiment.

Another distinction in our model is that resistance to this targeted therapy is not an irreversible phenotypic difference. Instead, under our assumptions, every cell had the capacity to evolve and regulate resistance. That is, resistance to the targeted therapy was within the normal cancer cell plasticity. That is, cell’s resistant strategy is a quantitative phenotypic trait that can be ramped up or down. This capability has been reviewed as a cause for therapeutic resistance in many cancers^48–51^. For instance, dynamic changes in chromatin structure (an epigenetic modification) were associated with transient drug-tolerant states in NSCLC^52^. In melanoma, one mechanism of resistance to BRAF inhibitors was attributed to the upregulation of EMT-gene signatures^53^. In various cancers, drug efflux properties are elevated in tumor cells by regulating gene expression to present as drug resistant, sensitive, or some middle ground between. Inhibiting the ability of tumor cells to adapt to their environment and circumvent drug-targeted pathways could be a promising strategy to combat therapeutic resistance. Studies have shown that targeting factors that drive tumor cell plasticity, such as signaling pathways and transcription factors can enhance the efficacy of anticancer drugs^54^. Whether or not therapy could regulate plasticity to a drug-sensitive state would be something interesting to consider as well.

We find that, when resistance is regulated by phenotypic plasticity, the outcomes of AT are the rates of phenotypic change from sensitive and resistance and vice versa. Increasing the rate of evolution when therapy was off significantly improved the efficacy of AT. Although in a previous study we showed that faster speeds of evolution reduced the improvement in TTP provided by AT, that was strictly assuming the rate of evolution towards resistance matched the rate of evolution towards sensitivity^27^. In the absence of strong selective forces against resistance this could be a useful strategy in controlling tumor burden.

A caveat to our study is that our models were unable to fully replicate the cyclic tumor growth dynamics in mice in the adaptive therapy group. This discrepancy may be attributed to the rapid fluctuations in tumor growth and regression, as well as a possible delayed response to therapy. In general, the model makes the assumption that drug-delivery is instant and does not accumulate. Incorporating a delay in drug response or known pharmacodynamics within the model framework could potentially address this issue and enhance model accuracy. Furthermore, it is crucial to acknowledge that while certain parameters were considered in our models, the complexity of tumor dynamics may require the inclusion of additional factors for a more comprehensive representation. One such limitation could be attributed to our mouse model used a single NSCLC cell line (H3122) xenografted into immunocompromised NSG mice. While this model captures certain features of ALK-positive NSCLC, it lacks the heterogeneity, immune interactions, and spatial constraints present in human tumors. Despite these limitations, our study provides valuable insights into the various aspects of AT efficacy and applicability.

In conclusion, the key lessons drawn from this study provide insights into the requirements for effective AT. Our results demonstrate that two main factors were found to hinder the effectiveness of AT in the NSCLC mouse model under investigation. Firstly, no density-dependent feedback was observed. Secondly, the evolutionary speed towards less resistance under no therapy was not larger than that towards greater resistance under therapy. These findings highlight the importance of thoroughly assessing and satisfying these two criteria in advance when planning an AT strategy.

## Materials and methods

### In vivo

Orthotopic mouse xenograft experimental protocols were approved by the Institutional Animal Care and Use Committee (IACUC) at the University of South Florida. Animals were maintained and evaluated under pathogen-free conditions in accordance with IACUC standards of care at the H. Lee Moffitt Cancer Center (Tampa, FL). NSCLC cell line H3122 was obtained from the Lung Cancer Center of Excellence Cell Line depository at Moffitt Cancer Center. H3122 cells were cultured in RPMI 1640 supplemented with 10% FBS (Invitrogen, Carlsbad, CA). The cell line was authenticated by STR analysis and tested negative for mycoplasma (PlasmoTest, InvivoGen, San Diego, CA). We used H3122 cells subcutaneously grafted into the right flank of immunodeficient NSG mice as an NSCLC model. 1* × *10^6^ cells were injected per animal. Tumors were measured twice a week (Tuesday and Friday) using a caliper. Two measurements were taken (longitudinal and lateral) to estimate relative tumor volume using the following formula: $\:3.14{lon}^{2}x\frac{lat}{6}$, where *lon* and *lat* are longitudinal and lateral values, respectively. Once the tumors reached 250-300mm^3^, mice were divided into three treatment groups: (1) continuous alectinib (LC Laboratories), (2) adaptive alectinib, and (3) control (no treatment). Each arm was represented by five mice. Alectinib was used in a dosage of 25 mg/kg (oral gavage) and delivered until tumors reached endpoint criteria of ≥ 2000 mm^3^ according to the following treatment protocols:

- Continuous: Alectinib was administered daily, five days a week (Monday-Friday).
- Adaptive: Alectinib was administered daily until the tumor volume decreased by ≥ 25% of the initial tumor size. Treatment was withheld until the tumor regains ≥ 50% of the difference between the initial size and the nadir after alectinib administration. The initial and nadir values of the tumor size were not static and were recorded specifically with each cycle of on-off therapy.

We chose size thresholds to better balance competition between resistant and sensitive cells while maintaining a lower tumor burden. We withheld therapy after a 25% shrinkage from initial size to reduce selective pressure on sensitive cells and resumed therapy when regrowth reached 50% of the drop from the initial size to the nadir.

### Mathematical models

We explored the results of our preclinical trial by modeling tumor growth dynamics in response to treatment as an evolutionary game. To do this, we employ multiple G-functions, which allow us to model the ecological dynamics of tumor growth and the evolutionary impact of cellular strategies (in our case, a resistance strategy) on these dynamics. We take into consideration two models of tumor growth, ultimately selecting one, and then create three G-functions, each with a different formulation for how resistance reduces drug efficacy.

We begin by modeling how the population size will grow over time in the absence of therapy. Instead of assuming a single model for tumor growth, we let our experimental data guide our choice between exponential and logistic growth, defined by the following equations:

$${\text{Exponential Growth}}:\,\,\frac{{dx}}{{dt}}=rx$$

$${\text{Logistic Growth}}:\,\,\frac{{dx}}{{dt}}=rx\left( {1 - \frac{x}{K}} \right)$$

where *r* is the intrinsic growth rate, *x* is the population size, and *K* is the carrying capacity. The exponential model depicts growth without limitations whereas the logistic model considers limits to growth by introducing density-dependence where per capita growth rates decline linearly with population size. At small population sizes (relative to carrying capacity) logistic growth mimics exponential growth. However, as the population size approaches carrying capacity, the change in population size slows. The incorporation of a carrying capacity introduces competition for resources and space between individuals within a population. We tested whether exponential or logistic population growth best explained our observed in vivo tumor growth by fitting each growth model to data from the control arm of the experiment where no treatment was given.

Next, we model how the presence of the drug suppresses cell proliferation. We consider three different ways to model mortality as a function of resistance:

1 $$\:\mathrm{M}\mathrm{o}\mathrm{r}\mathrm{t}\mathrm{a}\mathrm{l}\mathrm{i}\mathrm{t}\mathrm{y}\:\mathrm{M}\mathrm{o}\mathrm{d}\mathrm{e}\mathrm{l}\:1:\mu(v)=\:\frac{m}{1+bv}$$

2 $$\:\mathrm{M}\mathrm{o}\mathrm{r}\mathrm{t}\mathrm{a}\mathrm{l}\mathrm{i}\mathrm{t}\mathrm{y}\:\mathrm{M}\mathrm{o}\mathrm{d}\mathrm{e}\mathrm{l}\:2:\mu(v)=\:{me}^{-bv}$$

3 $$\:\mathrm{M}\mathrm{o}\mathrm{r}\mathrm{t}\mathrm{a}\mathrm{l}\mathrm{i}\mathrm{t}\mathrm{y}\:\mathrm{M}\mathrm{o}\mathrm{d}\mathrm{e}\mathrm{l}\:3:\mu(v)=\:{m(1-v)}^{b}$$

where *m* is the drug-induced death rate, *b* scales the efficacy of a resistant trait, and *v* is the focal cell’s resistance strategy. Equation 1 is adopted from Cunningham et al.^55^. In this context, therapy is likened to predation, and the model is constructed around the likelihood of prey encountering predators and the effectiveness of prey vigilance in minimizing mortality. Equation 2 is our formulation of cell mortality where we assume drug effectiveness decreases exponentially with resistance. Equation 3 is a generalized form of the tumor growth and inhibition (TGI) model^56^. In the formulation of each *µ*(*v*), the drug-induced death rate (*µ*) decreases as the strategy increases but at varying magnitudes determined by *b* (Fig S4). When *b* does not scale the value of resistance (*b* = 1), *µ*(*v*) declines linearly, such that model 3 requires less resistance to elicit a greater impact on *µ*(*v*) than the other models. At greater values of *b*, the dynamics of model 2 and model 3 become similar.

The volumetric experimental data were converted to cell density based on the estimate that 1*mm*^3^ = 10^6^ cells^57^. Parameter estimation for each model was conducted using the lmfit package in Python. Optimal parameter values were determined by minimizing the least squared difference between the calculated number of cells and model predictions. By fitting the model to the data, we now have parameter sets that represent each mouse. We use these parameter sets to conduct in silico trials, simulating different treatment protocols to determine if alternative strategies would extend progression-free survival.

## Supplementary Information

Below is the link to the electronic supplementary material.

Supplementary Material 1

Supplementary Material 2

## Data Availability

The data used in this research are provided in the supplementary information files.

## Code availability

The code used for modeling and data analysis was developed in Python and is publicly available on Zenodo at https://doi.org/10.5281/zenodo.17237109.

## Declarations

### ARRIVE statement

All procedures were conducted under the authority of the institutional ethical review committees (University of South Florida) and conducted in accordance with the ARRIVE guidelines.
