# Supplementary material for: Density dependence and evolvability limit adaptive therapy in non-small cell lung cancer mouse model
Source: Sci Rep. 2026 Aug 4;16:33742. doi: 10.1038/s41598-026-63987-x (PMC13694224; doi:10.1038/s41598-026-63987-x)
Supplement: Supplementary file 1 [file 41598_2026_63987_MOESM1_ESM.docx]

**Supplementary figures**

Supplementary Figure 1. Fits of each of the different models to the continuous therapy group data. Fixed values are *r* = 0*.*076 and *m* = 1. The shaded grey area represents when treatment was given. Models were fit to data assuming there were no weekend drug holidays. Each colored line represents a different model fit to the data. Due to the close agreement of all models, most lines overlap.

Supplementary Figure 2. Fits of each of the different models to the AT group data. Fixed values are *r* = 0*.*076 and *m* = 1. The shaded grey area represents when treatment was given. Each colored line represents a different model fit to the data. The models fail to capture fluctuations in data that may be the result of the on/off therapy switching protocol.

Supplementary Figure 3. Simulations of AT compared to continuous therapy. Treatment response dynamics of three AT protocols and continuous therapy tested *in silico* using parameter values determined by each model for a specific mouse (from the fix *m* approach). These dynamics are shown as the percent change in number of cells over time. Simulations corroborate preclinical results demonstrating the inferiority of AT to continuous therapy in extending TTP.

Supplementary Figure 4. Drug-induced death rate (*m*) for each model with varying values of the magnitude of resistance benefit (*b*). *µ*(*v*) determines how effective the drug is at suppressing tumor cell growth. We consider three different representations of how drug-induced mortality declines as a function of a cell’s resistance strategy(2, 2, 3. Each model varies in terms of the rate of change in drug efficacy with respect to changes in resistance. This is scaled by the magnitude of resistance benefit (*b*) such that when b increases, less resistance is required to reduce drug efficacy. In each model *m* = 1.
